# The cellular and molecular mechanisms of tissue repair and regeneration as revealed by studies in *Xenopus*

**DOI:** 10.1002/reg2.69

**Published:** 2016-10-28

**Authors:** Jingjing Li, Siwei Zhang, Enrique Amaya

**Affiliations:** ^1^ Division of Cell Matrix Biology and Regenerative MedicineSchool of Biological SciencesFaculty of BiologyMedicine and HealthUniversity of ManchesterManchesterM13 9PTUK

**Keywords:** limb regeneration, scar‐free wound healing, tail regeneration, tissue regeneration

## Abstract

Survival of any living organism critically depends on its ability to repair and regenerate damaged tissues and/or organs during its lifetime following injury, disease, or aging. Various animal models from invertebrates to vertebrates have been used to investigate the molecular and cellular mechanisms of wound healing and tissue regeneration. It is hoped that such studies will form the framework for identifying novel clinical treatments that will improve the healing and regenerative capacity of humans. Amongst these models, *Xenopus* stands out as a particularly versatile and powerful system. This review summarizes recent findings using this model, which have provided fundamental knowledge of the mechanisms responsible for efficient and perfect tissue repair and regeneration.

## 1 INTRODUCTION

A prominent question in biomedical research is how organisms respond to injuries and ultimately restore the morphological and functional integrity of tissues and organs, thus ensuring their survival. Humans and most other mammals fail to achieve scar‐free healing or to regenerate complex tissues as adults (Gurtner, Werner, Barrandon, & Longaker, 2008). In contrast, various non‐mammalian vertebrates retain the capacity to heal in a scar‐free manner and to regenerate various organs and appendages even as adults (Seifert & Maden, 2014). For example, fish can regenerate their fins and heart and urodele amphibians can heal wounds perfectly and regenerate a range of complex tissues and organs, including limbs, tails, lenses, and retina (Godwin, 2014). While the ultimate goal in regenerative medicine is to improve wound healing and regeneration in human patients, research on other animal model systems, especially on those that have efficient repair and regeneration abilities, promises to provide invaluable information on the molecular and cellular basis of these processes, which will pave the way toward more efficient and effective treatments that will entice our tissues to repair and regenerate better (Godwin, 2014; Seifert & Maden, 2014).

Amongst the various model systems that have been exploited for investigating the mechanisms of scar‐free healing and appendage regeneration is the anuran amphibian *Xenopus laevis* and its diploid relative *Xenopus tropicalis*. These frogs heal epidermal wounds without scar formation throughout embryonic and larval stages, and like urodele amphibians (e.g. newts, axolotls, and salamanders) they are able to regenerate limbs, tails, and lens at the larval stages (Beck, Izpisúa Belmonte, & Christen, 2009). However, unlike urodeles, which maintain regeneration capacity throughout life, post‐metamorphic *Xenopus* froglets lose their ability to fully regenerate their limbs (Godwin & Rosenthal, 2014). This stage‐dependent regenerative ability provides a powerful model for investigating the progressive loss of regenerative ability through ontogeny and also provides an excellent assay system for identifying mechanisms that prolong regenerative capacity (Beck et al., 2009; Lin, Chen, & Slack, 2013).

Prior to its exploitation as a model system for wound healing and tissue regeneration research, *Xenopus* has enjoyed a long history as a powerful and highly tractable system for the study of embryonic development. The key advantages of this system include *ex utero* development, which enables ready observation and manipulation of embryos at all stages of development; easy husbandry; and controllable induction of ovulation at any time of year, resulting in the production of large numbers of eggs (Amaya, 2005). In addition, *Xenopus* has an extensive array of genomic and genetic tools (reviewed in Harland & Grainger, 2011), including a published genome (Hellsten et al., 2010), extensive expressed sequence tag libraries (Gilchrist et al., 2004), transgenic protocols and reagents (Kroll & Amaya, 1996; Love et al., 2011b), and advanced genetic editing tools (Ishibashi, Cliffe, & Amaya, 2012; Nakayama et al., 2013).

In addition to its value as an experimental embryological system, *Xenopus* also provides a tractable and powerful system for investigating the mechanisms of tissue repair and regeneration. The large‐sized and easy‐to‐culture *Xenopus* oocytes have been used to study single‐cell wound healing, a fundamental process that shares many features in common with more complicated multicellular tissue and organ repair mechanisms (Sonnemann & Bement, 2011). Furthermore, the blastula stage embryo with thousands of cells (termed blastomeres) can be used to study multicellular scar‐free wound healing (Davidson, Ezin, & Keller, 2002; Li, Zhang, Soto, Woolner, & Amaya, 2013; Soto et al., 2013). Finally, research on the tadpole and later stages can be explored to investigate more complex tissue repair mechanisms, such as tail, limb, and lens regeneration (reviewed in Beck et al., 2009).

Here we summarize recent findings in both wound healing and tissue regeneration in *Xenopus*, and we highlight the value and potential of this system for elucidating key fundamental mechanisms that permit efficient scar‐free wound healing and appendage regeneration, with implications for regenerative medicine.

## 2 SINGLE‐CELL WOUND HEALING IN *XENOPUS* OOCYTES

**Figure 1:**
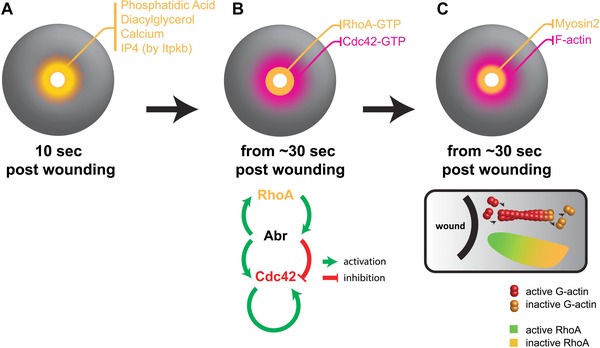
**Single‐cell wound responses**. (A) Immediate signals, including calcium, IP4, and others, are produced in a gradient at the wound edge, upstream of the cytoskeletal signaling. (B) From 30 sec post wounding onwards, small Rho GTPases Cdc42 and RhoA are activated at the wound edge. Spatial patterning of the circumferential rings of Cdc42 and RhoA is regulated by Abr, a dual‐functional GAP/GEF, which separates active Cdc42 and active RhoA into an outer ring and an inner ring, respectively. (C) Cytoskeletal machinery in single‐cell wound closure. Myosin‐2 and F‐actin also accumulate circumferentially at the wound edge, and the closure of this actomyosin ring is driven by a centripetal gradient of RhoA activity (box). Reciprocally, this RhoA activity is also regulated by treadmilling of the actin filaments at the wound edge

Even before the advent of multicellular life, unicellular organisms would have encountered various forms of potential injuries from mechanical, predatory, or chemical insults. Such injuries would have provided strong selective pressures for the advent of rapid and efficient unicellular repair mechanisms. To this day, such repair mechanisms remain critical for the ability of cells to survive both mechanical stresses generated by normal physiological processes (skeletal and cardiac muscle contraction) and those arising from various injuries from the external environment (McNeil & Steinhardt, 1997, 2003). Single‐cell wounds, like multicellular wounds, trigger a rapid wound healing response, aimed at reconstituting the barrier function between the inside and outside of the cell. This is done by rapidly resealing the plasma membrane through rapid exocytosis of intracellular membrane vesicles (Miyake & McNeil, 1995; Terasaki, Miyake, & McNeil, 1997). Research using *Xenopus* oocyte single‐cell wound healing assays revealed the participation of F‐actin and myosin‐2, two cytoskeletal components extensively involved as force‐generating machineries in cell movement and rearrangement, in single‐cell wound healing (see Fig. 1) (Bement, Mandato, & Kirsch, 1999; Mandato, Weber, Zandy, Keating, & Bement, 2001). Bement and colleagues also showed that a contractile zone of F‐actin and myosin‐2 forms at the wound circumference within seconds post wounding, promoting the constriction of the membrane at the wound margin (Bement et al., 1999; Mandato et al., 2001). By exploiting the benefit of the large size and the availability of high‐resolution live imaging techniques in the *Xenopus* oocyte system, researchers have been able to visualize the dynamics and spatial organization of key molecular players, such as the activation state of the small Rho GTPases, Cdc42 and RhoA, which underlie the formation and function of the contractile actomyosin array at the wound margin (Benink & Bement, 2005). Using this experimental system, it has also been possible to show that the closure of the actomyosin array is driven by centripetal gradients (i.e. towards the center of the wound) of Rho and Cdc42 activity (Burkel, Benink, Vaughan, Dassow, & Bement, 2012). Rho and Cdc42 are preferentially activated at the wound edge and inactivated away from the trailing edge away from the wound. Moreover, these gradients of Rho and Cdc42 are regulated by the contraction of myosin‐2 and the turnover of F‐actin, revealing a complex two‐way regulation of the power‐generating cytoskeleton and its upstream regulators (Burkel et al., 2012). Studies using the *Xenopus* oocyte wound healing system have also identified a dual‐functional protein, Abr, in coordinating the spatiotemporal activation of Rho and Cdc42 and consequentially the reorganization of the cytoskeletal machinery at the wound edge (Vaughan, Miller, Yu, & Bement, 2011). These findings have led to a mathematical model that is able to simulate the single‐cell wound healing process and, moreover, predict cellular responses under different patterns of injuries (Simon, Vaughan, Bement, & Edelstein‐Keshet, 2013).

Because the activation of Rho and Cdc42 is a robust response in single‐cell wound healing, researchers have endeavored to identify the molecules that act upstream of these small Rho GTPases during and/or following injury. It has been known for a long time that Ca^2+^ influx is required for Rho GTPase activation (Benink & Bement, 2005), and it was discovered recently that de novo synthesis and transport of different lipids to different domains at the wound site are also correlated with spatial organization of Rho activity and cytoskeletal machinery (Vaughan et al., 2014). The lipid diacylglycerol accumulates in a zone circumferential to the wound, and by acting through two antagonizing downstream factors, protein kinase β and η, regulates the activation of Rho and Cdc42 (Vaughan et al., 2014). Additional findings using *Xenopus* oocytes have identified the lipid kinase inositol‐trisphosphate 3‐kinase B (Itpkb), and its enzymatic product inositol 1,3,4,5‐tetrakisphosphate (InsP_4_), as essential regulators of single‐cell wound healing (Soto et al., 2013). Overexpression of Itpkb or application of InsP_4_ is able to enhance the activity of Rho and Cdc42, to enhance actin assembly at the wound edge, and to accelerate the speed of wound closure (Soto et al., 2013). The ability of Itpkb and especially its product InsP_4_ in accelerating wound healing makes it a potential target for improving the speed of acute and chronic wound healing in patients. In addition to the identification of wound healing promoters, a small molecule screen was carried out in the *Xenopus* oocyte wound healing system aimed at identifying molecules that alter the speed of healing (Clark et al., 2012). Greatly facilitated by the copious availability of oocytes from *Xenopus* females, their large size and tractability, two small molecules, Sph1 and Sph2, were found to downregulate Rho activation and impair single‐cell wound healing using this system (Clark et al., 2012). Taken together, *Xenopus* oocytes provide a tractable and powerful system for uncovering the molecular and cellular mechanisms responsible for single‐cell wound healing.

## 3 MULTICELLULAR WOUND HEALING IN *XENOPUS* EMBRYOS

**Figure 2:**
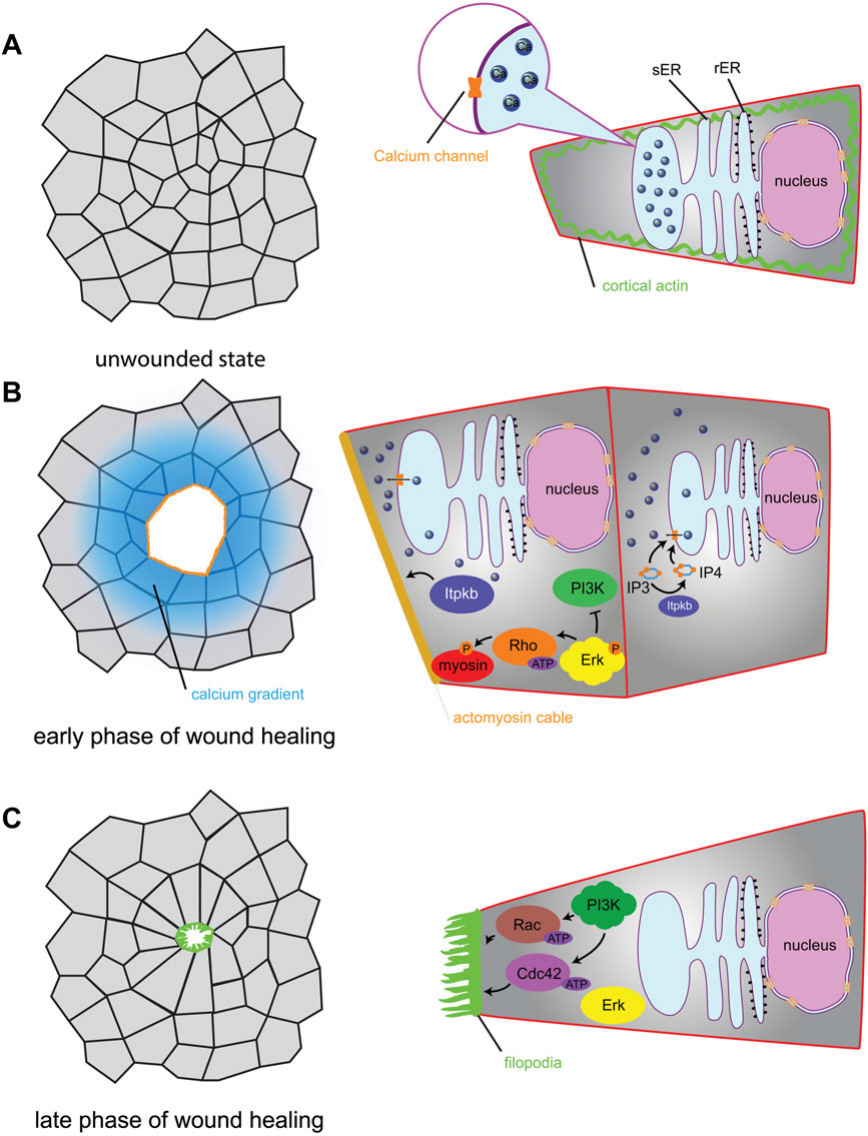
**Stages of multicellular wound healing**. (A) In an unwounded epithelium, calcium ions are stored in the network of smooth endoplasmic reticulum (ER) and cell shape is maintained by cortical actin. Blue beads, calcium ions. (B) At early stages post wounding, calcium ions are released from the ER storage through calcium channels. The opening of the calcium channels is promoted by both IP3 and the product of Itpkb, IP4. The wave of calcium release propagates planarly across several rows of epithelial cells, mobilizing a larger region of the epithelial sheet for reepithelialization. On the other hand, cortical actin in leading edge cells undergoes reorganization and forms a contractile actomyosin cable at the wound edge. Itpkb regulates the accumulation of F‐actin, whereas Erk signaling regulates myosin‐2 activation, mediated by active RhoA. PI3K activity is inhibited at this stage. (C) Closure of the wound at a later stage is driven by filopodial zippering at the leading edge. PI3K signaling is active, and through Rac and Cdc42 transforms early‐stage actomyosin cable to filopodial protrusions

Compared with single‐cell wound healing, which mainly consists of repairing disrupted membrane and constriction of an actomyosin array, multicellular wound healing involves simultaneous mobilization of a sheet of cells and subsequent collective movement (reviewed by Sonnemann & Bement, 2011). Thus, unlike single‐cell wounds, multicellular wound healing requires the coordination of both intracellular and intercellular signal transduction pathways and cell behaviors for successful wound repair. *Xenopus* embryos and larvae provide an excellent system to investigate the molecular and cellular mechanisms underpinning multicellular scar‐free wound healing. First the embryos can be produced in large numbers, and their large size and external development facilitate manipulating and observing wound healing processes. Research using this system has shown that multicellular wound closure shares several mechanisms with those seen in single‐cell wounds, such as a critical role for calcium influx, local activation of Rho GTPases at the wound margin, and formation of an actomyosin array, albeit on a multicellular scale (Stanisstreet, 1982; Clark et al., 2009) (Fig. 2). Constriction of the multicellular actomyosin array closes the wounded area, and intriguingly, in line with the finding in single‐cell wound healing, overexpression of Itpkb or application of InsP_4_ also enhances actomyosin assembly and wound contraction, implicating a shared molecular and cellular continuum in single‐cell and multicellular wound responses (Soto et al., 2013).

As is the case during single‐cell wound healing, calcium also acts upstream of Rho GTPase activation and reorganization of the cytoskeleton in multicellular wounds (Clark et al., 2009). However, Ca^2+^ signaling in multicellular wound healing is more complex in that it involves not only Ca^2+^ influx in the injured cells but also subsequent Ca^2+^ wave propagation from cell to cell traveling from the injury site, thereby mobilizing nearby uninjured cells for collective cell movement. Very little is known about the mechanisms that drive the propagation of Ca^2+^ signaling across the epithelium, although a recent finding showed that Itpkb and its product InsP_4_, besides enhancing Rho activity and actin assembly, also facilitate Ca^2+^ propagation across the epithelial tissue from the site of injury (Soto et al., 2013). Although it has been known from studies in other systems that both the actomyosin contraction and filopodial zippering are mechanical forces that help repair the wound (Wood et al., 2002), a recent study in *Xenopus* embryonic epithelium described a mechanism that temporally coordinates the function of these two distinct force‐generating machineries, thereby facilitating efficient embryonic wound healing (Li et al., 2013). This work showed that an early activation of Erk signaling initiates Rho activation and myosin‐2 phosphorylation, which in turn triggers actomyosin constriction for a quick phase of wound closure. Later on, PI3K signaling takes over, activating Rac and Cdc42, and the mode of cell motility is transformed to filopodial zippering to seal the wound edges (Li et al., 2013). Intriguingly, coordinated actomyosin‐based contraction also participates in the rapid neuroepithelial wound healing in the developing *Xenopus* brain, which expels damaged neuroepithelial cells from the brain, thereby protecting the tissue from further cell death (Herrgen, Voss, & Akerman, 2014). This contraction is initiated by ATP released from damaged cells and propagated as a calcium wave induced by purinergic receptors (Herrgen et al., 2014).

Tailbud stage embryos heal epithelial wounds in a scar‐free manner and in a comparable timescale to blastula stage embryos (Yoshii, Noda, Matsuzaki, & Ihara, 2005; Fuchigami, Matsuzaki, & Ihara, 2011). Thus, studying the cellular and molecular mechanisms of wound healing at these later stages adds to the growing repertoire of accessible stages for experimentation using *Xenopus* embryos. The tailbud stages are of particular interest, as these are the stages when inflammatory cells begin to respond to injuries (Costa, Soto, Chen, Zorn, & Amaya, 2008; Chen et al., 2009), and therefore these are the earliest stages when one can begin investigating the role of inflammation during the healing process. Furthermore, one can use these stages, as well as the slightly later tadpole stages, in combination with the establishment and use of transgenic lines, to investigate the mechanisms by which inflammatory cells are recruited to the site of injury and respond to infections (Smith, Kotecha, Towers, Latinkic, & Mohun, 2002; Love et al., 2011b; Paredes, Ishibashi, Borrill, Robert, & Amaya, 2015).

## 4 WOUND HEALING IN *XENOPUS* EMBRYOS, FROGLETS AND ADULTS

The mature skin of the post‐metamorphic *Xenopus* froglet and adult has a highly comparable histology with that of the mammalian skin, containing a layered epidermis and a spongy dermis underneath (Kawasumi, Sagawa, Hayashi, Yokoyama, & Tamura, 2013; Haslam et al., 2014). Unlike adult mammalian wound healing, which generally results in scar formation, wound healing in *Xenopus* froglets is scarless (Yokoyama et al., 2011). However, this capacity declines as froglets age, such that *Xenopus* adults heal wounds in a manner that results in scar‐like tissue (Bertolotti, Malagoli, & Franchini, 2013). It has long been noted that scarring and regenerative capacity are inversely related and, as such, scarring is inhibitory to regeneration (Harty, Neff, King, & Mescher, 2003). Changes in both the innate and adaptive immune systems have long been suggested to be responsible for this transition between regenerative capacity and scarring, both within the lifetime of an organism and between organisms (Harty et al., 2003; Mescher, Neff, & King, 2016). Indeed, the maturation state of the immune system and the activation of thymus post wounding have been correlated with increased incidence of scarring as *Xenopus* adults age (Franchini & Bertolotti, 2014). Thus, this age‐related change in the capacity for scar‐free healing, which largely correlates with decreased regenerative capacity, can be exploited to investigate the critical changes responsible for the switch from scar‐free to scarring events.

Even though, as *Xenopus* adults age, their propensity for scarring increases, wound healing still proceeds remarkably well in these organisms. Frogs produce many substances, including antimicrobial compounds, which are either not present or are present at much lower concentrations in the mammalian skin (Zasloff, 1987; Berkowitz, Bevins, & Zasloff, 1990). Indeed some of these compounds present in amphibian skin have been shown to promote wound healing (Lipsky, Holroyd, & Zasloff, 2008; Mashreghi et al., 2013; Di Grazia et al., 2015). Intriguingly, another research direction has recently been established that exploits a comparative approach by which *Xenopus* skin explants and human skin explants are used side by side, as *ex vivo* organ culture systems, to identify compounds or treatments that improve wound healing (Meier et al., 2013). In this study, thyrotropin‐releasing hormone, a hypothalamic regulator of thyroid hormone production that is abundant in frog skin, was found to stimulate migration, proliferation, and differentiation of keratinocytes in both *Xenopus* and human skin wounds, thus promoting wound healing (Meier et al., 2013). This pilot assay with *Xenopus* and human *ex vivo* skin cultures revealed conserved wound healing responses between these two species and opened new avenues for efficient testing of novel compounds or applications in wound healing research aimed at facilitating the translation of wound promoting mechanisms from amphibians to humans.

## 5 TAIL REGENERATION IN *XENOPUS*

**Figure 3:**
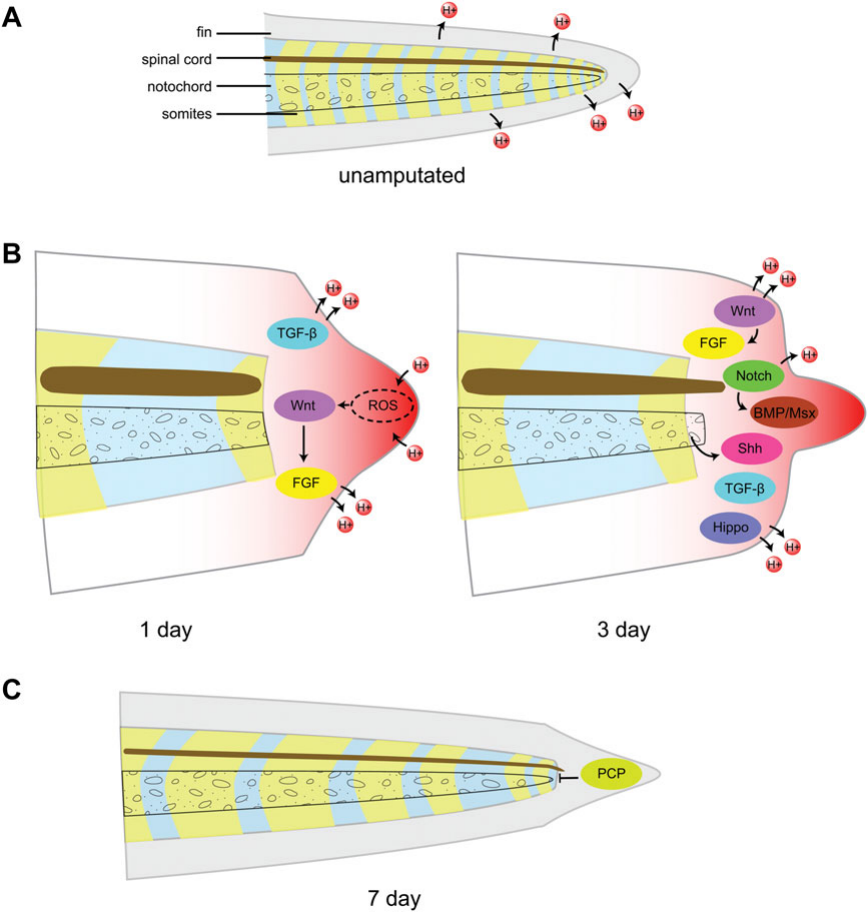
**Stages of tadpole tail regeneration**. (A) A *Xenopus* tadpole tail is composed of a number of axial structures including the spinal cord, notochord, and somites. An unamputated tail is in a polarized state, sustained by V‐ATPase pumps in the skin. (B) After amputation, wounded tail is depolarized and simultaneously reactive oxygen species (ROS) are produced at the amputation site. Downstream targets of the ROS include Wnt and FGF, and a number of other signaling pathways are required for successful regeneration such as Shh, TGF‐β, BMP, Notch, and Hippo pathways. V‐ATPases are also upregulated at this stage to repolarize the skin. (C) A fully functional tail is regenerated 7 days after amputation. The growth and termination of a regenerating tail are regulated by PCP signaling. BMP, bone morphogenetic protein; FGF, fibroblast growth factor; TGF‐β, transforming growth factor β; PCP, planar cell polarity

The *Xenopus* tadpole tail comprises various axial tissues, such as a spinal cord, notochord, somites, vasculature, and skin. These tissues are able to regenerate, resulting in a fully restored and functional tail 7−14 days post‐amputation (Love et al., 2011a; Chen, Love, & Amaya, 2014; Love, Ziegler, Chen, & Amaya, 2014) (Fig. 3). Tadpole tail regeneration follows three overlapping stages: an early phase, dominated by scar‐free healing and inflammation; an intermediate phase, dominated by the initiation of proliferation and the formation of the regenerative bud; and a late phase, when clear differentiation of tissues ensues (Love et al., 2011a). Intriguingly, regenerative capacity is stage‐dependent, in that only tadpoles younger than stage 45 or older than stage 48 are capable of regeneration, while tadpoles between stages 46 and 47 are refractory to regeneration (Slack, Beck, Gargioli, & Christen, 2004). The reason for this refractory period remains unclear. However, the period coincides with two important physiological transitions in the tadpole. One is the transition in nutritional sources from maternal yolk stores to food intake and digestion. Thus the refractory period coincides with a major change in metabolism, which may lead to a transient period of limited nutritional availability, required to feed the regenerative process. Another change that occurs during this period is maturation of the immune system, including the development of the adaptive immune system. Indeed, the refractory period can be inhibited by immunosuppression (Fukazawa, Naora, Kunieda, & Kubo, 2009), suggesting that the refractory period may be caused by changes in immunity. However, it remains unclear why older tadpoles regain full regenerative capacity, despite having an increasingly mature immune system. To this end, much remains to be learned to explain fully why the transitory refractory stage exists in the tadpole, and whether the mechanisms that underpin the refractory phase are relevant to why mammals lack full regenerative capacity.

In order to gain insight into the molecular and genetic mechanisms responsible for tadpole tail regeneration, a transcriptomic analysis was carried out during the three phases of tail regeneration, which revealed remarkable changes in gene expression in relation to inflammation and metabolism (Love et al., 2011a, 2014). For example, there is a significant and sustained increase in the expression level of several genes encoding enzymes associated with the production of reactive oxygen species (ROS), including NAD kinase, which phosphorylates NAD+ to NADP+, and glucose‐6‐phosphate dehydrogenase, which generates NADPH from NADP+ (Love et al., 2011a, 2013). NADPH is then used as a substrate for various NADPH oxidases, which generate superoxide and eventually other ROS (Bedard & Krause, 2007). Coincident with the increased expression in the genes encoding these metabolic enzymes, it was found that tail amputation is also associated with an increased and sustained production of ROS, throughout the regenerative response; and if this sustained ROS production is attenuated, using either pharmacological or genetic approaches, tail regeneration does not proceed (Love et al., 2013). Intriguingly, the increased ROS level appears to facilitate growth factor signaling, which is essential for tail regeneration (Love et al., 2013).

More recently, another study compared changes in gene expression profiles in injured spinal cords isolated from regenerative (tadpole) and non‐regenerative (post‐metamorphic froglet) stages (Lee‐Liu et al., 2014). The study found extensive transcriptome changes associated with stress response, metabolism, cell cycle, development, inflammation, and neurogenesis. Interestingly, the regenerative spinal cord takes a significantly shorter time to alter the gene expression level of amputation‐responsive transcripts, and the repertoires of regulated genes are significantly different from that found in non‐regenerative spinal cord tissues (Lee‐Liu et al., 2014). The study also identified many additional genes of unknown function, which also change their expression levels during spinal cord regeneration (Lee‐Liu et al., 2014). It is hoped that this system may provide an easily tractable model system for investigating the mechanisms that permit spinal cord regeneration in vertebrates (Lee‐Liu, Edwards‐Faret, Tapia, & Larraín, 2013; Muñoz et al., 2015).

While the mechanism of spinal cord regeneration is a fascinating and important problem in its own right, it is also of interest to investigate how the spinal cord coordinates the regeneration process as a whole. It has been known for nearly 200 years that appendage regeneration is nerve‐dependent (Todd, 1823; Singer, 1952; Kumar & Brockes, 2012). It has subsequently been shown that appendage regeneration in the *Xenopus* tadpole tail and limb is also nerve‐dependent (Filoni & Paglialunga, 1990; Taniguchi, Sugiura, Tazaki, Watanabe, & Mochii, 2008). In particular, removal of the spinal cord leads to significant defects in the patterning and growth of the tadpole tail during regeneration (Taniguchi et al., 2008). Furthermore, laser ablations aimed at generating more subtle injuries within the spinal cord at different anteroposterior positions also result in patterning and growth defects during tail regeneration (Mondia et al., 2011). Much work remains to uncover the mechanisms by which the spinal cord coordinates the growth and patterning of the tadpole tail during regeneration.

A signaling pathway that has often been associated with growth regulation and size control in various tissues, organs, and organisms is the Hippo pathway (Yu, Zhao, & Guan, 2015). It is thus perhaps not surprising that this pathway has also been shown to play a critical role in the control of the growth of the tadpole tail during regeneration (Hayashi et al., 2014). Another pathway involved in the growth control and/or termination of growth during development and regeneration is the planar cell polarity (PCP) pathway (Beane, Tseng, Morokuma, Lemire, & Levin, 2012).

Besides the Hippo and PCP pathways, several other signaling pathways have been shown to play important roles during tail regeneration, such as the Wnt, Notch, BMP, FGF, Shh, and TGF‐β pathways (Beck, Christen, & Slack, 2003; Beck, Christen, Barker, & Slack, 2006; Ho & Whitman, 2008; Lin & Slack, 2008; Taniguchi, Watanabe, & Mochii, 2014). Outstanding questions with regard to the regulation of these signaling pathways include, for example, what is the source of the signals? What are their upstream activators? How are the various pathways coordinated during the complex regeneration process?

On the cellular level, a question that has preoccupied researchers for many years is: which cells give rise to the nascent tissue during regeneration and where do they come from? Work performed primarily on urodele amphibians has suggested that blastema cells (mesenchymal stem cells) are the cells that give rise to the nascent tissues in the regenerating appendage, and furthermore that these cells come at least partly from dedifferentiation from adult mesenchymal cells near the amputation site (Brockes & Kumar, 2005; McCusker, Bryant, & Gardiner, 2015). Much interest has been devoted to understanding the biology of blastema cells, as they retain positional memory (cell identity associated with proximal−distal positions within the appendage) and are able to self‐organize. One question that has interested scientists is whether blastema cells are pluripotent or whether they exhibit lineage restriction. Another key question is whether all blastema cells arise from dedifferentiation or whether some arise from activated quiescent stem cell pools. Answering these questions required the advancement of tools which would allow lineage‐tracing experiments to be carried out over long periods of time. Such tools were finally developed in the mid‐1990s with the development of transgenic technologies in amphibians, and given that *Xenopus* was the first amphibian where such technologies were developed (Kroll & Amaya, 1996), it is not surprising that the first experiments using transgenic lines to investigate the origin and potency of blastema cells during tail regeneration were done in *Xenopus* tadpoles (Gargioli & Slack, 2004; Slack et al., 2004). Interestingly, these experiments showed that the regenerating tissues arise primarily from lineage‐restricted precursors/stem cells and little or no transdifferentiation or metaplasia is evident (Gargioli & Slack, 2004). Furthermore, the authors found strong evidence that the regenerating muscle arises from the resident stem cell pool of satellite cells rather than from dedifferentiated myofibrils, which are more commonly seen during urodele tail or limb regeneration (Lo, Allen, & Brockes, 1993; Gargioli & Slack, 2004; Tanaka, 2008; Rodrigues, Christen, Martí, & Izpisúa Belmonte, 2012). Overall the authors concluded that appendage regeneration in *Xenopus* follows mechanisms more similar to those seen during mammalian tissue renewal than those operating during urodele appendage regeneration. Interestingly, more recent findings, using similar approaches of employing transgenic lines to assess the origin and potency of cells in the blastema in axolotls and mammals, have shown that tissue regeneration generally follows lineage restriction, suggesting that dedifferentitation to a pluripotent blastema state is relatively uncommon during appendage regeneration in both amphibians and mammals, and metaplasia in urodeles occurs only in a relatively small subset of tissues, such as within connective tissues (e.g. dermis being able to form both cartilage and tendon) (Kragl et al., 2009; Rinkevich, Lindau, Ueno, Longaker, & Weissman, 2011).

## 6 LIMB REGENERATION IN *XENOPUS*

**Figure 4:**
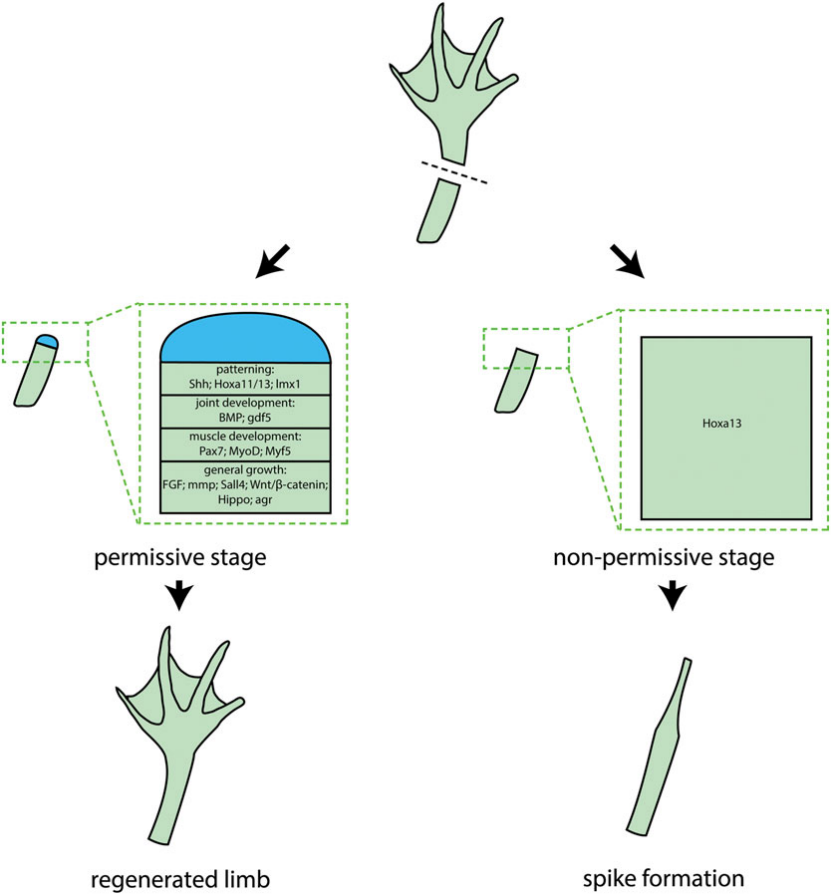
***Xenopus* as a model to compare regenerative and non‐regenerative limbs**. Before metamorphosis, *Xenopus* froglets are capable of regenerating amputated limbs. Listed are required genes and pathways in different processes of limb regeneration, including growth, patterning, joint and muscle development. Post‐metamorphic froglets enter a non‐permissive stage of limb regeneration when amputated limb can only grow into a spike instead of a restored limb. Many of the upregulated genes and activated pathways in the permissive stage are not properly expressed or activated at the non‐permissive stage

Unlike tail regeneration, limb regeneration in *Xenopus* has an ontogenic decline, whereby regenerative capacity decreases with age. This makes *Xenopus* an excellent model for elucidating the mechanisms that may promote regenerative capacity in non‐regenerative stages/organisms (Lin et al., 2013). As in other cases of regeneration, there is considerable interest in investigating whether limb regeneration in *Xenopus* simply recapitulates the mechanisms of limb development. To this end, it is very important to understand the mechanisms of limb development in *Xenopus*, so that proper comparisons to the mechanisms of regeneration can be performed. In contrast to other commonly used models of limb development, such as the chick and mouse, much less is known about the mechanisms of limb development and morphogenesis in *Xenopus*, even though pioneering studies on this model were done nearly 60 years ago (Tschumi, 1957; Keenan & Beck, 2016). Nevertheless, the few studies that have been done on limb development in *Xenopus*, using modern molecular approaches, have suggested that the mechanisms of limb formation in this model are largely conserved with those found in other tetrapods (Christen & Slack, 1998; Keenan & Beck, 2016). To this end, the spatiotemporal expression patterns of most genes known to play critical roles in limb development in the chick and the mouse are similarly expressed during *Xenopus* limb development (McEwan, Lynch, & Beck, 2011; Wang & Beck, 2014; Keenan & Beck, 2016), and indeed most of the molecular players involved during limb development partake as well during limb regeneration (Fig. 4).

One could ask, what is the use of yet another model system for investigating the mechanisms of limb regeneration if several powerful ones are available amongst the urodele amphibians? While urodele amphibians represent the species with the greatest capacity for limb regeneration amongst the vertebrates (McCusker et al., 2015), anuran amphibians such as *Xenopus* represent a unique model organism which is capable of limb regeneration at the early limb bud stages of development, but this regenerative capacity decreases as the limb development proceeds such that in the post‐metamorphic froglet only a hypomorphic spike regenerates after amputation (Dent, 1962; Muneoka, Holler‐Dinsmore, & Bryant, 1986; Beck et al., 2009). As such, *Xenopus* is intermediate between the full regenerative ability of urodeles and no regenerative capacity, as seen in birds and mammals, including humans. Thus, *Xenopus* tadpoles can be explored to investigate the mechanisms that permit limb regeneration during the permissive stages, versus those that impede it during the non‐permissive stages, within the same model organism. Furthermore, one can explore this system in order to investigate the molecular and cellular mechanisms that will prolong or promote regeneration during normally non‐permissive stages. A particularly poignant example of this sort of study was published by Gufa Lin and colleagues in 2013. In that monumental piece of work, the authors performed a series of very careful and technically demanding experiments to ask whether transplantation of blastema‐derived progenitor cells from regenerative stage tadpoles into post‐metamorphic froglet amputated limbs would be able to enhance the regenerative capacity of amputated limbs of non‐regenerative post‐metamorphic froglets. The answer was yes, but to get optimal enhancement of regeneration required that the transplanted cells had active Wnt/β‐catenin signaling and for the transplanted cells to be placed near sources of Shh, FGF10, and thymosin β4 (Lin et al., 2013). These findings suggest that it might be possible, in the future, to enhance the regenerative capacity of mammalian limbs using cell transplantation approaches, but any success will probably depend on a combination of factors, including the origin and age of the transplanted progenitor cells and what signaling pathways are active within them and in their vicinity.

## 7 CONCLUSIONS

*Xenopus* is a versatile and highly tractable system for research in wound healing and complex appendage regeneration. One emphasis of future research with this system is to obtain more mechanistic insights of the molecular and cellular bases of repair and regeneration, using the advanced imaging and genomic tools that have been developed in this system in the past decade. Another emphasis of future research is comparative studies between different stages of *Xenopus* with different wound healing and regeneration competence, as well as comparing healing and regenerating processes in *Xenopus* and mammals. Both directions have been touched on in previous work, but more detailed examination is still needed. It is hoped that these fundamental understandings of wound healing and regeneration in *Xenopus* will soon lead to the development of treatments aimed at improving healing, reducing scarring, and promoting functional regeneration of tissues in humans who have experienced traumatic injuries.
